# LncRNA Gm44981 modulates EZH2–H3K27me3–p21 axis to suppress mesangial cell senescence and kidney aging

**DOI:** 10.1080/0886022X.2026.2628471

**Published:** 2026-02-11

**Authors:** Jie Li, Limin Wei, Fanfan Gao, Lu Zeng, Hongli Jiang, Lei Chen

**Affiliations:** a Dialysis Department of Nephrology Hospital, The First Affiliated Hospital of Xi’an Jiaotong University, Xi’an, Shaanxi, China; b Department of Nephrology, People’s Hospital of Zhengzhou University, He’nan Provincial People’s Hospital, He’nan Provincial Key Laboratory of Kidney Disease and Immunology, Zhengzhou, China

**Keywords:** Kidney aging, LncRNA, *Gm44981*, p21, EZH2, H3K27me3

## Abstract

Aging imposes significant influence in alteration of organ structure, function, and susceptibility to disease. Long non-coding RNAs (lncRNAs) are frequently dysregulated in the aging kidney, however, the key lncRNAs and associated mechanism involved in regulating kidney aging remains poorly investigated. Herein, we performed unbiased whole transcriptome sequencing on the kidney tissue samples from young and aging mice. The results of differentially expressed lncRNAs among two groups revealed that *Gm44981*, a 1943 bp lncRNA, was down regulated in the aging kidney. Fluorescence *in situ* hybridization (FISH) assay showed that *Gm44981* was mainly located in the nucleus of glomerular mesangial cells (MCs). Functional experiments showed that overexpression of *Gm44981* in MC cells significantly promoted cell proliferation capacity. Specifically, overexpression of Gm44981 was associated with increased H3K27me3 level and enhanced enrichment of *EZH2* at the *Cdkn1a* promoter region, which correlated with the suppression of *Cdkn1a* expression and attenuated MCs senescence. Together, our findings highlighted the crucial roles of lncRNA *Gm44981* in regulation of glomerular MCs senescence and provided novel targets for aging therapy.

## GRAPHICAL ABSTRACT

**Figure:**
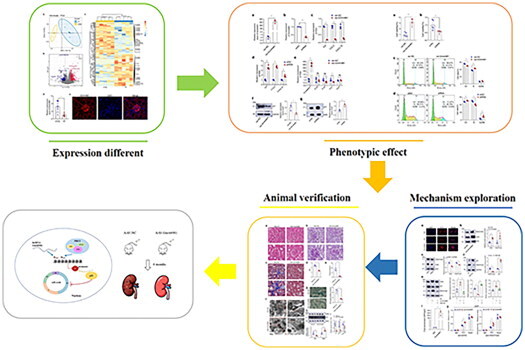

## Introduction

With the advancement of social civilization and medical treatment, the life expectancy of human beings has increased significantly, and the prevalence of age-related diseases such as dementia, hypertension, stroke, chronic kidney disease (CKD) and myocardial infarction has also increased remarkably, which poses a series of challenges to the life quality and mental health of the elder people, as well as clinical practices [1,2].

The human aging process is characterized by a progressive functional decline as well as macroscopic and microscopic histological alterations in a variety of organ systems, especially in the kidney because of the extremely active metabolic signature [3]. Although aging per se does not cause kidney injury, the molecular, structural, and functional changes alongside kidney aging is a crucial inducer and high-risk factor of increased incidence of acute kidney injury (AKI) and CKD. The alteration in glomerular structure during the aging process, such as thickening of glomerular basement membrane and expansion of mesangial matrix, were the most important risk factors leading to irreversible reduction in kidney function and even fibrosis [4,5]. Cellular senescence is one of the common occurrences during the development of chronic diseases in the body and aging process. The glomerular mesangial cells (MCs), one of the most important compositions in the glomerular, are crucial in maintaining mesangial matrix homeostasis, regulating glomerular filtration rate, and keeping normal glomerular function. The kidney aging process is usually accompanied by the MCs function dysregulation, such as the abnormal proliferation of MCs and ECM deposition [6]. Dissecting the dynamic molecular characteristics of the aging MCs contributes to clarify the cellular signaling mechanism of aging and explore potential targets for slowing or even reversing kidney aging.

Long non-coding RNA (lncRNA), a kind of RNA with length more than 200 nucleotides, exhibited diverse functional effects, including epigenetic regulation, (post) transcriptional gene regulation, and compartmentalization. Increasing evidence highlighted the vital role of lncRNAs in regulation of developmental process and disease pathogenesis, including cancer, inflammation, and aging [7–9]. Recent research also revealed that lncRNAs play crucial regulatory role in the pathogenesis of aging related kidney disease, such as diabetic nephropathy, glomerular disease, and renal fibrosis [10,11]. However, these studies mainly focused on the lncRNAs associated with kidney aging and kidney disease progression, the core lncRNAs and associated mechanism in slowing or even reversing kidney aging remained indistinct.

While EZH2-mediated H3K27me3 modification is a well-established epigenetic mechanism implicated in cellular senescence across various tissues, its regulatory specificity in kidney aging—particularly within glomerular mesangial cells—remains poorly understood. Previous studies have largely focused on EZH2’s broad roles in aging-related transcriptional silencing [12,13], yet the upstream regulators that direct *EZH2* to specific genomic loci in a cell type– and context-dependent manner are still emerging. LncRNAs have recently been recognized as key molecular guides that recruit epigenetic complexes to distinct gene promoters, thereby fine-tuning senescence programs [14]. However, whether and how lncRNAs orchestrate *EZH2* targeting to senescence-associated genes in kidney aging is largely unexplored. Notably, lncRNA–EZH2 interactions have been implicated in other renal pathologies, such as renal fibrosis and glomerular injury [15,16], underscoring their broader significance in kidney biology.

In this study, we identify lncRNA *Gm44981* as a novel kidney-aging–downregulated transcript that predominantly localizes in glomerular mesangial cells. We demonstrate that *Gm44981* specifically recruits *EZH2* to the *Cdkn1a* promoter, enhancing H3K27me3 deposition and epigenetically repressing *Cdkn1a* expression, thereby attenuating mesangial cell senescence. Our findings not only reveal *Gm44981* as a key regulator of mesangial cell aging but also provide a mechanistic paradigm in which a tissue-enriched lncRNA directs *EZH2* activity to a specific senescence-related gene locus, offering a novel layer of specificity in epigenetic regulation of kidney aging.

## Methods

### Animal samples

A total of 5 3-month-old (young) and 5 24-month-old (old) C57BL/6J mice kidney tissues were collected in this study. All mice were raised in a standard environment, and had free access to food and water. These collected tissues were frozen in liquid nitrogen and stored at −80 °C. The SAMP8 male mice (4-month-old) were obtained from the Department of Laboratory Animal Science, Peking University Health Science Center, Beijing, China. The strain is registered under the MGI (Mouse Genome Informatics) accession ID: MGI: 2160863. To established the *Gm44981* overexpressed model, SAMP8 male mice (4-month-old) were received tail vein injected with AAV-Gm44981 or AAV-nc as a negative control (synthesized by Genechem, Shanghai, China). Each mouse received a single tail vein injection of a total dose of 1 × 10^12 v.g. Four months after AAV9 injection, observation under fluorescence microscope was performed to confirm that *Gm44981* was expressed in the mouse kidneys. Briefly, the frozen sections of kidney tissues were placed in a dry box and dried at temperature for 30–45 min away from light. After the sections were firmly attached to the slides, the sections were washed three times in PBS for 10 min each time. After the embedding agent was cleaned and removed, the excess PBS on the slides was blotted with filter paper, and the slides were sealed with sealer. Fluorescence microscopy was used to observe and collect data. After 4 months of AAV9 administration (at the age of 8 months), the mice were anesthetized with urethane by intraperitoneal injection at a dose of 750 mg/kg and then euthanized, and kidney tissues were harvested for subsequent analyses (Supplementary Figure S1).

The study was approved by the Animal Ethics Committee of Xi’an Jiaotong University (Shaanxi, China) (No. 2018-G-164). All methods were carried out in accordance with the animal ethics guidelines and regulations. This study was carried out in compliance with the ARRIVE guidelines.

### RNA-sequence analysis

RNA-sequence analysis for the expression of lnRNAs was performed by Hangzhou Lianchuan Biotechnology CO., Ltd. (Hangzhou, China), using whole transcriptome sequencing. RNA sequencing was performed on the Illumina Novaseq™ 6000 platform with a 2 × 150 bp paired-end strategy. After raw read filtering with Cutadapt and quality control with FastQC, the high-quality clean reads were aligned to the mouse reference genome (GRCm38) using Hisat2 and Bowtie2. Transcripts were then assembled and their expression levels were quantified as FPKM using StringTie. Differential expression analysis of mRNAs and lncRNAs was conducted using DESeq2/edgeR, with transcripts satisfying an absolute fold change >2 and a false discovery rate (FDR *q*-value <0.6), considered statistically significant. The coding potential of lncRNAs was assessed using CPC and CNCI. The accession number for the data reported in this paper is Gene Expression Omnibus database GEO: GSE154223.

### Cell culture

The mouse kidney glomerular mesangial cell lines SV40 MES 13 (MCs) were purchased from National Collection of Authenticated Cell Cultures (Shanghai, China) and were cultured in DMEM medium (Gibco, Thermo Fisher Scientific, USA) containing 10% fetal bovine serum (FBS) at 37 °C with 5% CO_2_ in cell incubator. This cell line was originally established by transfection of primary mesangial cells from SJL mice with an SV40 plasmid and has been widely used as a model for glomerular mesangial cell biology [17,18]. Upon receipt, cell identity was routinely confirmed by morphology observation under phase-contrast microscopy (exhibiting a typical stellate or spindle-shaped, non-overlapping growth pattern), and cells were used at low passages (p. 5–15) to maintain phenotypic stability.

### Reagents and transfection

To overexpression of *Gm44981*, overexpression plasmid of *Gm44981* (oe-Gm44981) and negative control (oe-NC) were designed and synthesized by Genechem (Shanghai, China). siRNA and negative were purchased form GenePharma (Shanghai, China). The sequence of siRNA as follow: siNC: sense 5′-UUCUCCGAACGUGUCACGUTT-3′, antisense 5′-UUCUCCGAACGUGUCACGUTT-3′; siRNA: sense 5′-GGAGACACUGUGCCUGCUUTT-3′, antisense 5′-AAGCAGGCACAGUGUCUCCTT-3′. Cells were seeded into 6-well plates incubated 18–24 h before transfection. Plasmid and siRNA transfection were performed using Lipofectamine 2000 (Invitrogen, USA) according to the manufacturer’s instructions and collected the cells after transfection 48h for further analysis. For functional assays, cells were transfected with plasmid or siRNA for 48 h before further analyses. A schematic summary is provided in Supplementary Figure S2.

### RNA extraction and quantitative real-time PCR (qRT-PCR)

Total RNA was extracted from kidney or cultured cells by Trizol Reagent (Takara, Dalian, China) according to the manufacturer’s protocols. The concentration and purity of RNA were checked, and OD260/280 ratio between 1.8 and 2.0 is qualified. The cDNA was synthesized using reverse transcriptase (Takara, Dalian, China). Stepone Real-Time PCR System (Bio-Rad) was used to carry out all the PCR reaction. *GAPDH* was used as the endogenous controls. Triplicates were performed for each gene. Relative expression levels were calculated for unknown genes by using 2^-ΔΔCt^. Each lncRNA number and primer sequence are shown in Table 1.

**Table 1.** Primer sequences used in qRT-PCR.

Name	Forward primer (5′-3′)	Reverse primer (5′-3′)
*Gm44981*	CGGCCACAGAGCACAACTTCC	AGCACGTTCACCACAGAATCATCC
*Cdkn2b*	AATCCAGGTCATGATGATGGG	CTGCTCTTCAGCCAAGTCTAC
*Cdkn2a*	GCTCAACTACGGTGCAGATTC	GCACGATGTCTTGATGTCCC
*Cdkn1a*	ATGTCCAATCCTGGTGATGTC	GAAGTCAAAGTTCCACCGTTC
*Cdkn1b*	TTTAATTGGGTCTCAGGCAAAC	CCCTTTTGTTTTGCGAAGAAGA
*IL-6*	CTCCCAACAGACCTGTCTATAC	CCATTGCACAACTCTTTTCTCA
*CCL2*	TTTTTGTCACCAAGCTCAAGAG	TTCTGATCTCATTTGGTTCCGA
*CXCL10*	CAACTGCATCCATATCGATGAC	GATTCCGGATTCAGACATCTCT
*GAPDH*	GGTTGTCTCCTGCGACTTCA	TGGTCCAGGGTTTCTTACTCC

### Cell proliferation assays

Cell proliferation was measured using cell counting kit-8 (CCK8) assay. 48 h after transfection, 90 ul of new medium and 10ul of CCK-8 solution was added to the cells (Beyotime, C0042). Cells were incubated 1–4 h at 37 °C in 5% CO_2_ and measured at 450 nm by universal microplate reader (Bio-Tek, USA). The cell-free wells were set as blank control group. Experiments were performed in triplicated.

### Flow cytometric analysis

Cells were plated in 6-well plates the day before transfection. After 48 h of transfection, the cells were trypsinized, centrifuged in 1000 rpm, 5 min. Then cells were fixed in 70% ethanol at 4 °C at least 4 h. After being centrifuged, RNase A and propidium iodide (PI) staining solution were added to the cells, and the cells then incubated for 30 min at room temperature in the dark. The stained cells were resuspended and analyzed using a ACEA NovoCyte (Biosciences, USA).

### Western blotting assay

Cells were washed with ice-cold PBS and then 120 μL RIPA buffer (Heart, China) was added for cell lysis. Then the cells were collected, centrifuged, and quantified. Loading buffer was added and boiled for denaturation. Thirty micrograms of proteins were separated by 15% SDS-PAGE gel (Heart, China) and electro transferred to PVDF membranes (ThermoFisher, USA). Then membranes were blocked with 5% nonfat milk. The membranes were incubated with specific primary antibodies: anti-p21 (Abcam, ab109199), anti-H3K27me3 (Abcam, ab6002), Cdk2 (Abcam, ab32147), anti-actin (Proteintech, 6008-1-Ig), and anti-GAPDH (Proteintech, 60004-1-Ig) overnight at 4 °C. After washing with TBST, membranes were incubated with a secondary antibody for 1h at room temperature. Protein bands were visualized using a chemiluminescence kit (Millipore, USA). The expression of actin or GAPDH was used to normalize protein.

### *Fluorescence* in situ *hybridization (FISH)*

The cells were fixed with 4% paraformaldehyde for 10 min, then washed with PBS, and permeabilized with pre-cooled permeation solution, and then washed with PBS three times. Probes were mixed with pre-prepared hybridization buffer, and then samples were incubated overnight in hybridization buffer at 37 °C. After rinsing with hybridization buffer at 37 °C for 15 min, the samples were rinsed three times quickly at room temperature and stained with DAPI staining solution. Finally, images were captured using a confocal microscope.

### RNA immunoprecipitation (RIP)

RNA immunoprecipitation (RIP) was used to measure the ability of *Gm44981* to bind *EZH2* by using the RIP kit (Millipore, USA) according to the manufacturer’s instructions. The cells were lysed with RIPA lysis in ice-bath for 5 min. A part of the cell extract was saved as an Input, and the remaining was incubated with IgG or anti-EZH2 (CST, 5246) for co-precipitation. The bead-protein complex was collected on a magnetic base. The RNA was extracted from co-precipitation and input samples after detachment from the bead using protease K. The expression of Gm44981 was determined using qRT-PCR.

### Chromatin immunoprecipitation (ChIP) assay

Chromatin immunoprecipitation (ChIP) was performed using the EpiQuik Chromatin Immunoprecipitation (ChIP) Kit (Epigentek, USA), according to the manufacturer’s protocols. Firstly, cell lysates were sonicated to generate chromatin fragments of 200–1000 bp. Then, the chromatin was immunoprecipitated using anti-EZH2 (CST, 5246), anti-H3K27me3 (Abcam, ab6002). qRT-PCR was performed to detect the expression of *Cdkn1a.* The primers targeting the mouse p21 promoter region (Chr17: 29309036–29309153, GRCm39) were: 5′-CTGCCTCTGCTCAATAATGTTTCT-3′ (forward), 5′-GGAATTCACCTTCACACAGGC-3′ (reverse).

### Histopathology

Mice renal tissue specimens were fixed in 10% paraformaldehyde solution overnight. Then, the sections were dehydrated, paraffin-embedded, and sectioned at 4-μm thickness. For HE staining, sections were dewaxed with xylene and then stained with hematoxylin and eosin according to standard histological procedures. PAS and Masson trichrome staining were performed using standard protocols. Images were captured by camera, and the area of Masson-stained positive images was calculated with Image J. At least 20 glomeruli from each kidney were selected and graded according to the following criteria: 0, no sclerosis; 1, <25% sclerosis in cross section; 2, 25–50% sclerosis; 3, 50–75% sclerosis; and 4, more than 75% sclerosis. The mean score per glomerulus in each kidney was determined as the glomerular sclerosis index (GSI).

For transmission electron microscopy analysis, kidney tissues were fixed in 2.5% glutaraldehyde and 4% paraformaldehyde solution for more than 2 h at 4 °C; then the tissues were rinsed in 0.1 M phosphate buffer; then the tissues were put into 1% osmium fixative for more than 2 h at 4 °C, rinsed again, the tissues were sequentially dehydrated in a series of gradients in 30% acetone, 50% acetone, 70% acetone, 90% acetone and 100% acetone. The tissues were immersed in embedding agent for 12 h or overnight. The tissues were embedded with epoxy resin and polymerized at high temperature (60–80 °C) to form an embedding block of moderate hardness; then it was made into semithin sections of 1 mm thickness, stained with toluidine blue, positioned under optical microscope, and the area to be conserved was selected for ultrathin sectioning; uranyl acetate (TEM stain) and lead citrate were stained for 15 min each. They were then dried and observed under transmission electron microscope.

### SA-β-gal staining

SA-β-gal activity was analyzed using an SA-β-gal staining kit (Beyotime, C0602) according to the manufacturer’s protocol. The area of positive staining was measured, and SA-β-gal-positive cells were calculated using Image-Pro Plus 6.0.

### Statistical analysis

The measurement data in accordance with the normal distribution were presented as the mean ± *SD* of three independent biological replicates, unless otherwise specified. Each biological replicate was performed with separately cultured and treated cell samples or tissues from different animals. Comparisons between two groups were analyzed by the unpaired Student’s *t*-test. For comparisons among more than two groups, one-way ANOVA followed by Bonferroni correction post-hoc test was applied. *p*-Value <0.05 was statistically significant. All calculations were carried out by using GraphPad Prism 8 (GraphPad Software, Inc., USA).

## Results

### The differentially expressed lncRNAs in old and young mouse kidney

**Figure 1.**
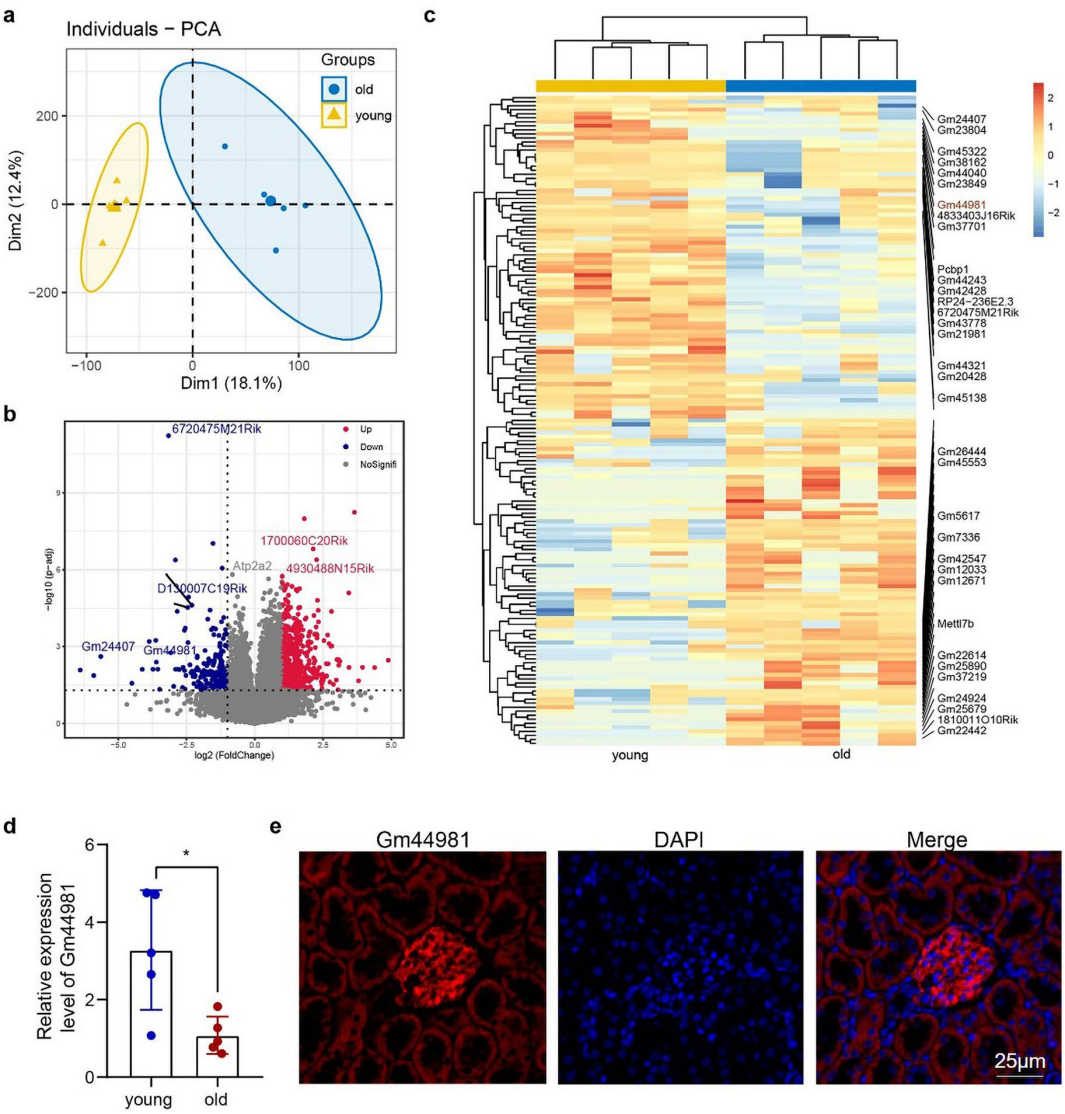
lncRNA profiling in aging mice kidney tissue. (a) Principal component analysis (PCA) plot of lncRNA expression profiles from young (3-month-old, yellow triangles, *n* = 5) and aging (24-month-old, blue circles, *n* = 5) mouse kidney tissues. Each symbol represents an individual biological sample. The x-axis (PC1) and y-axis (PC2) represent the first and second principal components, explaining XX% and YY% of the total variance, respectively. The shaded areas indicate the 95% confidence intervals for each group. (b) Volcano plot displaying the differential expression analysis. The x-axis shows the log2 fold change (aging/young). The y-axis shows the − log10 of the *p*-value (unpaired *t*-test). Gray dots represent lncRNAs not meeting significance thresholds. Red dots indicate significantly upregulated lncRNAs (log2 FC > 1, *p* < 0.05), and blue dots indicate significantly downregulated lncRNAs (log2 FC < −1, *p* < 0.05). The position of Gm44981 is highlighted with a green arrow. Horizontal dashed line: *p* = 0.05 threshold. Vertical dashed lines: |log2 FC| = 1 threshold (corresponding to 2-fold change). (c) Heatmap of the top Z-score normalized expression of differentially expressed lncRNAs (|fold change| > 2, *p* < 0.05) between young and aging kidneys. Rows represent individual lncRNAs, and columns represent individual samples. The color scale indicates expression levels from low (blue) to high (red). (d) qPCR demonstrated the expression of *Gm44981* between young and aging mice kidney tissue samples. Data are from independent biological replicates and are presented as mean ± *SD. n* = 5. *p < 0.05 *versus* young group. (e) FISH analysis for location of Gm44981 in mouse kidney tissue samples. Scale bar: 25 μm.

To dissect the key lncRNAs vital for kidney aging, the tissue samples from young mouse kidneys and aging mouse kidneys (*n* = 5) were used for the whole transcriptome sequencing. The principal component analysis (PCA) revealed significant difference of expression of lncRNAs between aging kidneys and young kidneys (Figure 1(a)). We identified 130 upregulated and 91 downregulated lncRNAs in the aging kidneys compared to young kidneys (Figures 1(b,c), *p*-value <0.05, fold change >2). Among the downregulated lncRNAs in aging kidney, *Gm44981* was found to be significantly expressed in young mouse kidney than in old mouse kidney (Figures 1(b,c)). Furthermore, the expression of *Gm44981* was validated to be downregulated in the aging kidney tissue by qPCR (Figure 1(d)). Taking advantage of the UCSC database (GRCm38/mm10) (http://genome.ucsc.edu/), we found that *Gm44981* was located on Chromosome 6: 91,711,336–91,713,278 and composed of 1 exon in mouse (Supplementary Figure S3(A)). The CPC (http://cpc2.cbi.pku.edu.cn/) analysis demonstrated that *Gm44981* didn’t have the protein coding capability (Supplementary Figure S3(B)). PyhloCSF was used to score the coding function, and the analysis results showed that *Gm44981* coding scores were all <0, which further proved that *Gm44981* had no protein coding ability (Supplementary Figure S3(C)). Immunofluorescence staining revealed that *Gm44981* was predominantly expressed in glomeruli (Figure 1(e)). In brief, lncRNA *Gm44981* was mainly expressed in glomeruli and downregulated in aging kidney compared with young kidney.

### Gm44981 *regulates MCs senescence through down-regulation of senescence-associated secretory phenotype (SASP) and cell cycle genes*

**Figure 2.**
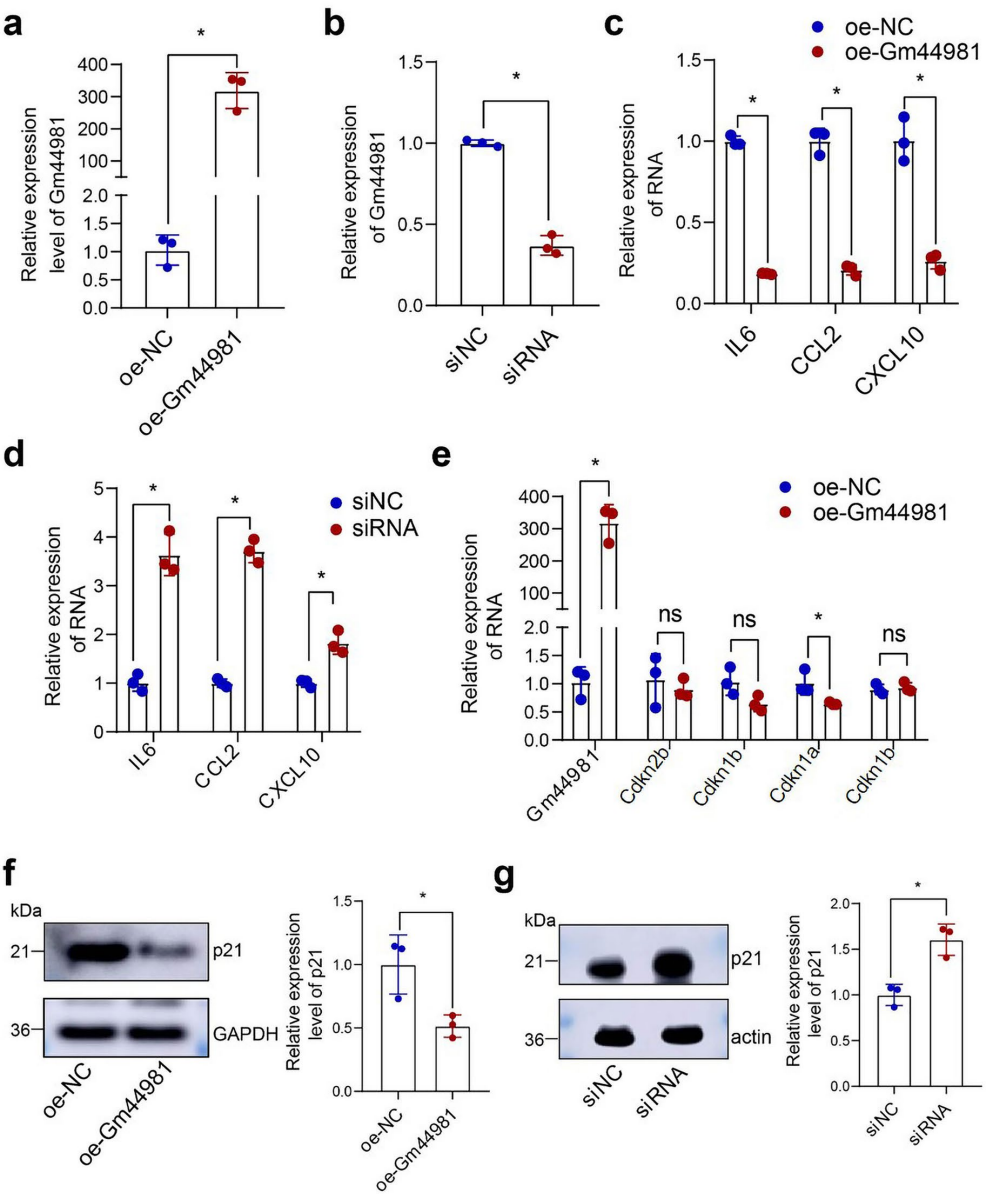
Gm44981 regulates MCs senescence. (a) qRT-PCR show that relative gene expression of *Gm44981* after MCs transfected with oe-Gm44981. Data are from independent biological replicates and are presented as mean ± *SD. n* = 3. *p < 0.05 *versus* oe-NC. (b) qRT-PCR show that relative gene expression of *Gm44981* after MCs transfected with siRNA. Data are from independent biological replicates and are presented as mean ± *SD. n* = 3. *p < 0.05 *versus* si-NC. (c) qRT-PCR showed that relative gene expression of *IL6*, *CCL2*, and *CXCL10* after MCs transfected with oe-Gm44981. Data are from independent biological replicates and are presented as mean ± *SD. n* = 3. *p < 0.05 *versus* oe-NC. (d) qRT-PCR showed that relative gene expression of *IL6*, *CCL2*, and *CXCL10* after MCs transfected with siRNA. Data are from independent biological replicates and are presented as mean ± *SD. n* = 3. *p < 0.05 *versus* si-NC. (e) qRT-PCR showed that relative gene expression of *Cdkn2b*, *Cdkn2a*, *Cdkn1a*, and *Cdkn1b* after MCs transfected with oe-Gm44981. Data are from independent biological replicates and are presented as mean ± *SD. n* = 3. *p < 0.05 *versus* oe-NC. (f) Western blotting analysis showed protein expression of p21 in MCs after transfection oe-Gm44981. Data are from independent biological replicates and are presented as mean ± *SD. n* = 3. *p < 0.05 *versus* oe-NC. (g) Western blotting analysis showed protein expression of p21 in MCs after transfection siRNA. Data are from independent biological replicates and are presented as mean ± *SD. n* = 3. *p < 0.05 *versus* si-NC. oe-NC represent negative control of overexpressed plasmid, oe-Gm44981 represent overexpressed plasmid.

The above results showed that *Gm44981* was specifically expressed in glomeruli, and the aging kidney exhibited a decreased expression level of *Gm44981* indicative of a potential protective role of *Gm44981* in kidney aging. Since mesangial cells are the main cellular component of the glomeruli, we next investigated whether Gm44981 regulates their senescence. To study the function of *Gm44981* in MCs, we conducted gain-of and loss-of function experiment in the MCs. Firstly, we transfected the overexpressed plasmid and siRNA into MCs, and checked the *Gm44981* expression to verify the efficiency of the overexpressed plasmid (oe-Gm44981) and siRNA (Figures 2(a,b)). We further investigated the expression of SASP genes (*IL6*, *CCL2*, and *CXCL10*), which are hallmark secretory factors produced by senescent cells. Interestingly, the results showed that the SASP genes were significantly downregulated in the oe-Gm44981 MCs group, while siRNA treatment increased the SASP genes expression in MCs dramatically (Figures 2(c,d)). This indicates that attenuation of cellular senescence by Gm44981 leads to a reduction in the SASP. To further explore the effects of Gm44981 on MCs senescence, the expression of cyclin dependent kinase inhibitors (CDKIs) genes such as *Cdkn2b*, *Cdkn2a*, *Cdkn1a*, *Cdkn1b* were measured. The qRT-PCR results showed that the expression of *Cdkn1a* was significantly decreased in MCs after transfected with overexpression plasmid of Gm44981 (Figure 2(e)). Additionally, the western blotting analysis verified *Gm44981* had significant influence on the regulation of p21 protein expression level (Figures 2(f,g)). These results are consistent with a role for Gm44981 in the regulation of MCs senescence, as evidenced by its correlation with down-regulation of SASP genes *IL6*, *CCL2*, and *CXCL10*, and cyclin dependent kinase inhibitor p21.

### Gm44981 *promotes MCs proliferation capacity*

**Figure 3.**
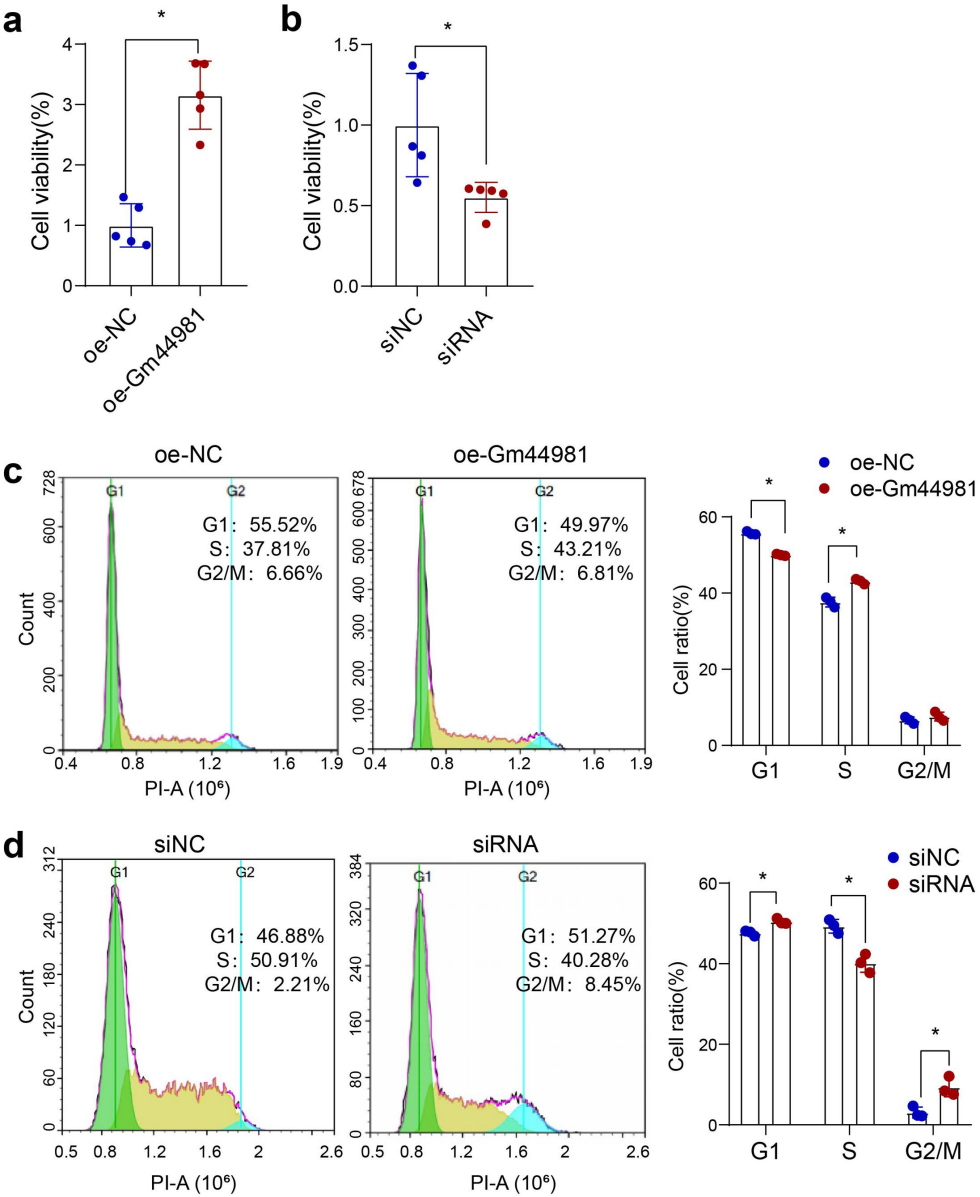
Gm44981 promotes MCs proliferation capacity. (a) CCK8 assay showed that the cell viability of MCs after transfection with oe-Gm44981. Data are from independent biological replicates and are presented as mean ± *SD. n* = 5. *p < 0.05 *versus* oe-NC. (b) CCK8 assay showed that the cell viability of MCs after transfection with siRNA. Data are from independent biological replicates and are presented as mean ± *SD. n* = 5. *p < 0.05 *versus* si-NC. (c) Flow cytometric analysis showed that cell cycle percentage of MCs after transfection with oe-Gm44981, the bar charts summarizing cell fractions in the G1, S, and G2 phases of the cell cycle. Data are from independent biological replicates and are presented as mean ± *SD. n* = 3. *p < 0.05 *versus* oe-NC. (d) Flow cytometric analysis showed that cell cycle percentage of MCs after transfection with siRNA, the bar charts summarizing cell fractions in the G1, S, and G2 phases of the cell cycle. Data are from independent biological replicates and are presented as mean ± *SD. n* = 3. *p < 0.05 *versus* si-NC. oe-NC represent negative control of overexpressed plasmid, oe-Gm44981 represent overexpressed plasmid.

Given the ability of *Gm44981* in regulating MCs senescence, we further explored whether *Gm44981* affects the proliferation capacity of senescent MCs. The CCK8 assay suggested that the expression level of *Gm44981* was highly associated with the cell viability, indicated that upregulation of *Gm44981* promoted the proliferation rate of MCs, and vice versa (Figures 3(a,b)). We further analyzed the cell cycle portion of MCs when receiving oe-Gm44981 and siRNA perturbation. Interestingly, the flow cytometry analysis revealed that the percentage of MCs with G1 phase was decreased whereas the S phase was increased when the MCs were transfected with overexpression plasmid of *Gm44981* (Figure 3(c)). Additionally, the transfection of *Gm44981* siRNA in MCs led to decrease percentage of S phase cells and increase percentage of cells in G1 phase and G2/M phase (Figure 3(d)). These results demonstrated that *Gm44981* played vital role in promoting MCs proliferation ability.

### Gm44981 *inhibited* Cdkn1a *expression in association with* EZH2 *enrichment at its promoter*

**Figure 4.**
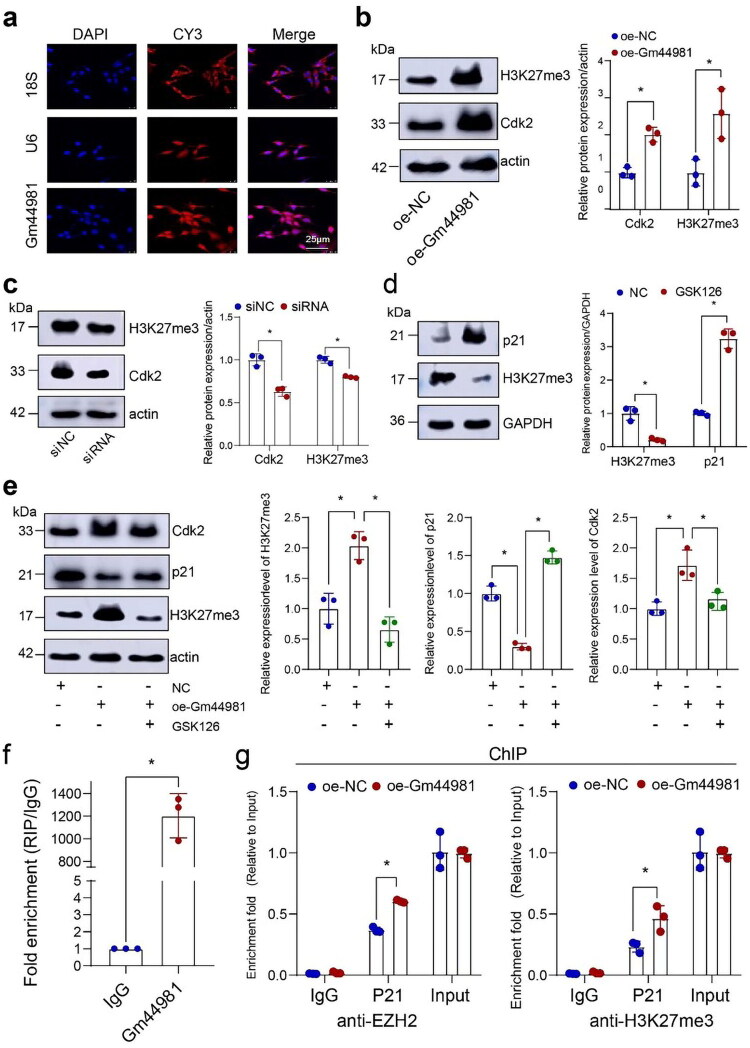
*Gm44981* inhibits *Cdkn1a* expression in association with *EZH2* enrichment at its promoter. (a) FISH analysis revealed the distribution of *Gm44981* in subcellular fraction of MCs. Scale bar, 25 μm. (b) Western blotting analysis showed expression of H3K27me3 and Cdk2 in MCs after transfection oe-Gm44981. Data are from independent biological replicates and are presented as mean ± *SD. n* = 3. *p < 0.05 *versus* oe-NC. (c) Western blotting analysis showed expression of H3K27me3 and Cdk2 in MCs after siRNA transfection. Data are from independent biological replicates and are presented as mean ± *SD. n* = 3. *p < 0.05 *versus* si-NC. (d) Western blotting analysis showed expression of p21 and H3K27me3 in MCs treated with GSK126. Data are from independent biological replicates and are presented as mean ± *SD. n* = 3. *p < 0.05 *versus* NC. (e) Western blotting analysis showed the expression of H3K27me3, p21, and Cdk2 protein after MCs transfected with oe-Gm44981. Data are from independent biological replicates and are presented as mean ± *SD. n* = 3. *p < 0.05 *versus* NC. (f) RIP assay of the enrichment of *Gm44981* to *EZH2* in MCs. Data are from independent biological replicates and are presented as mean ± *SD. n* = 3. *p < 0.05 *versus* IgG. (g) ChIP assays were conducted on P21 promoter regions using the indicated antibodies. Enrichment was determined relative to input control. Data are from independent biological replicates and are presented as mean ± *SD. n* = 3. *p < 0.05 *versus* oe-NC. oe-NC represent negative control of overexpressed plasmid, oe-Gm44981 represent overexpressed plasmid. *p < 0.05 *versus* negative control. oe-NC represent negative control of overexpressed plasmid, oe-Gm44981 represent overexpressed plasmid.

DNA methylation at age-associated CpG sites, which most likely occurred in the regulatory regions of genes involved in carcinogenesis, has been reported as a biomarker of aging and health [19]. According to previous research, the age-associated CpG sites are also bound by *EZH2*, a catalytic subunit of Polycomb Repressive Complex 2 (PRC2). PRC2 catalyzes H3K27me3, which can recruit DNA methyltransferases (DNMTs) to facilitate DNA methylation at these loci [20]. We further explored whether *Gm44981* could regulate the aging process through epigenetic regulation by influencing the H3K27me3 level. FISH results revealed that *Gm44981* was distributed in both the nucleus and cytosol, but mainly located in the nucleus (Figure 4(a)), which indicated that lncRNA *Gm44981* might involve in transcriptional regulation in nucleus [21–23]. The interaction probability analysis demonstrated a high score between *Gm44981* and *EZH2*, the RF and SVM score was 0.75 and 0.62, respectively. We examined the histone methylation levels in MCs transfected with overexpressed plasmid of *Gm44981* and found that the expression of H3K27me3 increased largely compared with negative control (Figure 4(b)). In contrast, knockdown of *Gm44981* showed the opposite effect (Figure 4(c)). These results indicated that *Gm44981* have significant influence in regulation of H3K27me3 levels. As we know, *EZH2*, the histone methyltransferase of PRC2 repressed gene transcription by catalyzing H3K27me3. Whether *Gm44981* could inhibit the expression of *Cdkn1a* through *EZH2* in kidney remains unknown. To functionally assess whether the EZH2-H3K27me3 axis is required for the effects of *Gm44981*, we treated MCs with the EZH2 methyltransferase inhibitor GSK126. As expected, GSK126 treatment markedly reduced global H3K27me3 levels and concomitantly increased p21 protein expression (Figure 4(d)). Critically, when *Gm44981*-overexpressing MCs were co-treated with GSK126, the reduction in p21 and the increase in Cdk2 mediated by *Gm44981* were largely abrogated (Figure 4(e)). This pharmacological intervention experiment indicates that the enzymatic activity of *EZH2* is necessary for *Gm44981* to exert its suppressive effect on p21 and its pro-proliferative outcome, providing functional support for the involvement of this epigenetic pathway. The RIP assay revealed that *Gm44981* significantly bound with EZH2 compared with IgG control (Figure 4(f)). To further address whether Gm44981 repressed P21 transcription through recruiting EZH2 to P21 promoters, we conducted ChIP analysis in Gm44981-overexpressed MCs. ChIP assay demonstrated that *Gm4498*1 overexpression increases the occupancy of *EZH2* and the deposition of H3K27me3 at the *Cdkn1a* promoter region. Together with the RIP data, these findings support a model where *Gm44981* is associated with *EZH2* and facilitates its epigenetic repressive function at the specific genomic locus of *Cdkn1a* (Figure 4(g)).

### *Overexpression of* Gm44981 *inhibited the kidney aging* in vivo

**Figure 5.**
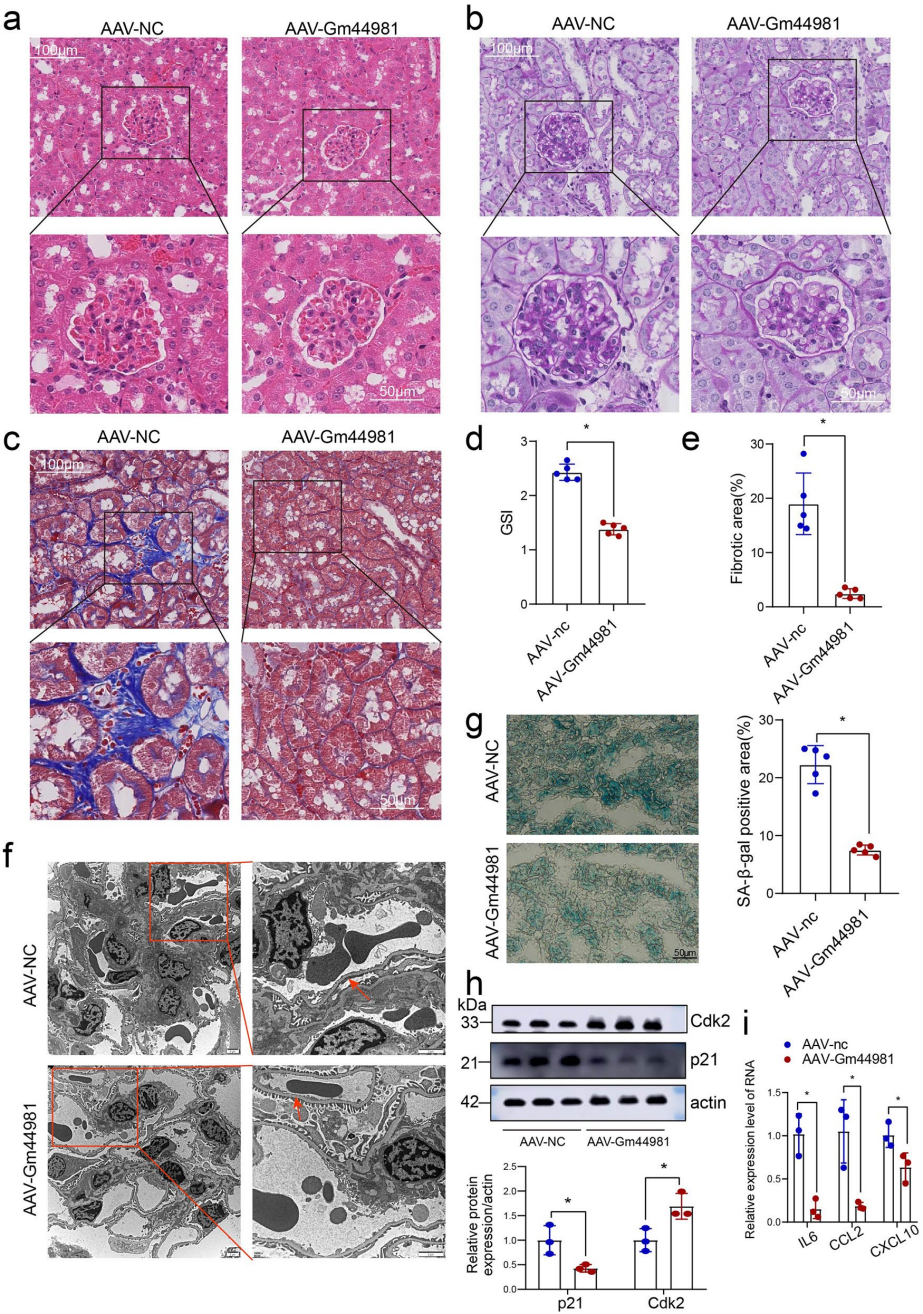
Overexpression of Gm44981 ameliorated aging-associated histopathological lesions in the kidney of SAMP8 mice. (a) H&E staining of SAMP8 mice renal tissue. Scale bar: 50 μm. (b) Masson staining of SAMP8 mice renal tissue. Scale bar: 50 μm. (c) PAS pathological staining of SAMP8 mice renal tissue. Scale bar: 50 μm. (d) Glomerular sclerosis index. Data are from independent biological replicates and are presented as mean ± *SD. n* = 5. *p < 0.05 *versus* negative control. (e) Graphical representation of the extent of fibrotic area after quantitative determination. Data are from independent biological replicates and are presented as mean ± *SD. n* = 5. *p < 0.05 *versus* negative control. (f) Transmission electron microscope detected pathological changes in the kidneys of SAM8 mice. Scale bar: 2 μm. (g) SA-β-gal staining of SAMP8 mice renal tissue. Data are from independent biological replicates and are presented as mean ± *SD. n* = 5. *p < 0.05 *versus* negative control. Scale bar: 50 μm. (h) Western blotting analyses showed p21 and Cdk2 expression in SAMP8 mice renal tissue. Data are from independent biological replicates and are presented as mean ± *SD. n* = 3. *p < 0.05 *versus* negative control. (i) qRT-PCR showed that the RNA expression level of IL-6, CCL2, and CXCL10 was significantly decreased in AAV-Gm44981 group compared to negative control group. Data are from independent biological replicates and are presented as mean ± *SD. n* = 3. *p < 0.05 *versus* negative control.

To further examine the effect of *Gm44981* on regulation of kidney aging *in vivo*, we established SAMP8 mice model with overexpression of AAV-Gm44981 by tail vein injection. Immunofluorescence was performed to confirm that *Gm44981* was expressed in the mouse kidneys (Supplementary Figure S4). Compared with control group, the renal indicators such as renal tubular atrophy score, renal interstitial fibrosis score, and mesangial matrix collagen deposition were alleviated in the AAV-Gm44981 group (Figures 5(a–e)). In the kidney of SAMP8 mice from the AAV-nc group, the glomerular mesangial region was hyperplastic, the podocyte foot was intermittently fused, and the filtration barrier was damaged. While in the AAV-Gm44981 group, the glomerular mesangial region hyperplasia was reduced, the podocyte foot was arranged orderly and filtration barrier structure was intact (Figure 5(f)). Compared with AAV-nc group, the positive area of SA-β-gal in the AAV-Gm44981 group was significantly reduced (Figure 5(g)). In addition, we detected the expression of aging related proteins and found that p21 expression was decreased and Cdk2 expression was increased in the renal tissues of the AAV-Gm44981 group (Figure 5(h)). An important feature of cell senescence was the appearance of SASP, which was consists of a series of cytokines, such as pro-inflammatory cytokines, such as *IL-1α*, *IL-1β*, and *IL-6*, growth factors such as TGF-β, GM-CSF, and HGF, chemokines, such as *CXCL-1/3/10* and matrix metalloproteases. Then, we detected the RNA expression level of SASP, the results showed that the expression of *IL-6*, *CCL2*, and *CXCL10* was significantly decreased in AAV-Gm44981 group compared to negative control group (Figure 5(i)). In all, these results suggested that *Gm44981* could inhibit renal aging *in vivo*.

## Discussion

The human being’s organ system aging and related disorders has been an international focus as life expectancy keeps increasing. Kidney is a highly metabolic organ system which serve important role in blood purification, and kidney aging induced renal function injury brings many propositions about health and quality of life in human beings [24,25].

In the process of kidney aging, various types of cells of glomeruli and renal tubules have undergone different degrees of physiological changes, although some of the damage is reduced by compensation, the decline in kidney function cannot be reversed [5,26]. Cellular senescence is a state of permanent cell cycle arrest characterized by marked metabolic activity and dramatic changes in cell morphology [27,28], which has significant influence on organ aging in humans. The MCs are one of the most important intrinsic cell compositions in the glomerular, and senescence within the MCs is directly related to the renal dysfunction. Therefore, understanding the molecular changes in MCs senescence process is critical for developing potential targets for slowing or even reversing kidney aging.

LncRNAs can act as molecular scaffolds and have been shown to guide epigenetic factors to chromatin structure, which can then regulate gene expression through chromatin modification, transcription, and posttranscriptional processing. Previous studies have highlighted the emerging importance of lncRNAs in the promotion of aging of multiple organs, including kidney [9,29–31]. Our study identifies lncRNA *Gm44981* as a novel suppressor of mesangial cell senescence, functioning through the EZH2–PRC2 epigenetic pathway. Our data are consistent with a model in which nuclear-enriched Gm44981 facilitates the enrichment of EZH2 and the repressive histone mark H3K27me3 at the promoter of the critical senescence gene p21, leading to its transcriptional silencing. This recruitment enhances the deposition of the repressive histone mark H3K27me3, leading to transcriptional silencing. This finding directly connects *Gm44981* to the epigenetic control of kidney aging, positioning it as a key regulator of the chromatin landscape in aging glomeruli.

The downstream effects of this epigenetic silencing converge on the core p21–Cdk2 aging axis. By repressing p21, a principal mediator of senescence, *Gm44981* alleviates the brake on Cdk2 activity. This action facilitates G1/S phase progression, counteracts G1 arrest, and promotes cellular proliferation. Furthermore, *Gm44981* overexpression effectively suppressed the cellular senescence hallmarks exemplified by the SASP, significantly reducing the expression of pro-inflammatory factors (*IL-6*, *CCL2*). Thus, *Gm44981* orchestrates a multi-faceted anti-senescence program by epigenetically repressing a central node in the aging network.

The quest to counteract age-related epigenetic drift has emerged as a frontier in aging research. Strategies such as using senolytics to clear senescent cells [32], modulating sirtuins to enhance genomic stability [33], and recalibrating epigenetic clocks [34,35] reflect a paradigm shift toward targeting the fundamental mechanisms of aging. Our discovery of *Gm44981* aligns perfectly with this conceptual framework. The age-associated decline of *Gm44981* represents a form of epigenetic drift in the kidney, and its restoration rectifies the aberrant H3K27me3 landscape at specific loci. Therefore, we propose *Gm44981* as a ‘candidate renal epigenetic modifier’—a naturally occurring lncRNA whose activity is integral to maintaining a youthful epigenetic and cellular state in the glomerulus.

The mechanistic insights from this study open exciting avenues for future therapeutic development. The success of AAV-mediated overexpression of *Gm44981 in vivo* highlights the potential of AAV-lncRNA therapeutics for age-related kidney diseases. More precise interventions could leverage CRISPR-dCas9 epigenetic editors fused to *EZH2* or demethylase domains to directly rewrite the epigenetic code at the p21 promoter or other senescence-related genes, offering a strategy for reversing glomerular aging at its epigenetic root. The primary goal of such interventions to restore mesangial cell homeostasis would be to halt or reverse the expansion of the mesangial matrix and preserve the glomerular filtration barrier, thereby mitigating the decline in renal function with age. While challenges in delivery and specificity remain, targeting key epigenetic modifiers like *Gm44981* represents a promising and innovative path toward achieving renal rejuvenation.

Our findings on the *Gm44981*–*EZH2*–p21 axis have significant implications beyond physiological aging, extending to several common chronic kidney diseases (CKD). In diabetic kidney disease (DKD), mesangial expansion is a hallmark pathological feature, and p21 activation is known to promote mesangial cell senescence and matrix overproduction, contributing to disease progression [36]. Our data suggest that the downregulation of Gm44981, leading to p21 derepression, could be a shared mechanism driving mesangial pathology in both aging and DKD. Similarly, there is extensive overlap of pathways during aging and CKD progression, and coordinated cellular senescence, coordinated by key regulators such as p21, is increasingly regarded as one of the common pathways [37]. The dysregulation of EZH2 and p21 we observed is also a common feature in aging, irrespective of the initial insult [37,38]. Therefore, the *Gm44981*–*EZH2*–p21 pathway we identified may represent a common molecular node linking various injurious stimuli to mesangial cell senescence and glomerulosclerosis, positioning it as a promising therapeutic target for a spectrum of age-related and progressive kidney diseases.

## Limitations and future perspectives

Notwithstanding, this study has limitations that point to important future directions. The association between Gm44981 and EZH2 at the p21 promoter requires further validation through direct interaction assays (e.g., RNA pull-down), and genetic rescue experiments are needed to definitively establish EZH2 as the indispensable mediator. While the SV40 MES13 cell line is a valuable model, it may not fully recapitulate primary mesangial cell biology, and additional senescence markers (e.g., p16INK4a, γH2AX) should be examined to specify the regulated pathway. Furthermore, whether Gm44981 influences EZH2 occupancy globally or preferentially at senescence-related loci remains to be elucidated *via* genome-wide epigenetic profiling. Finally, although systemic AAV9 delivery ameliorated renal aging, the relative contribution of mesangial cells *versus* other renal cell types warrants investigation using cell-type-specific tools. Addressing these points will further elucidate how Gm44981 fine-tunes epigenetic regulation in renal aging.

**Figure 6.**
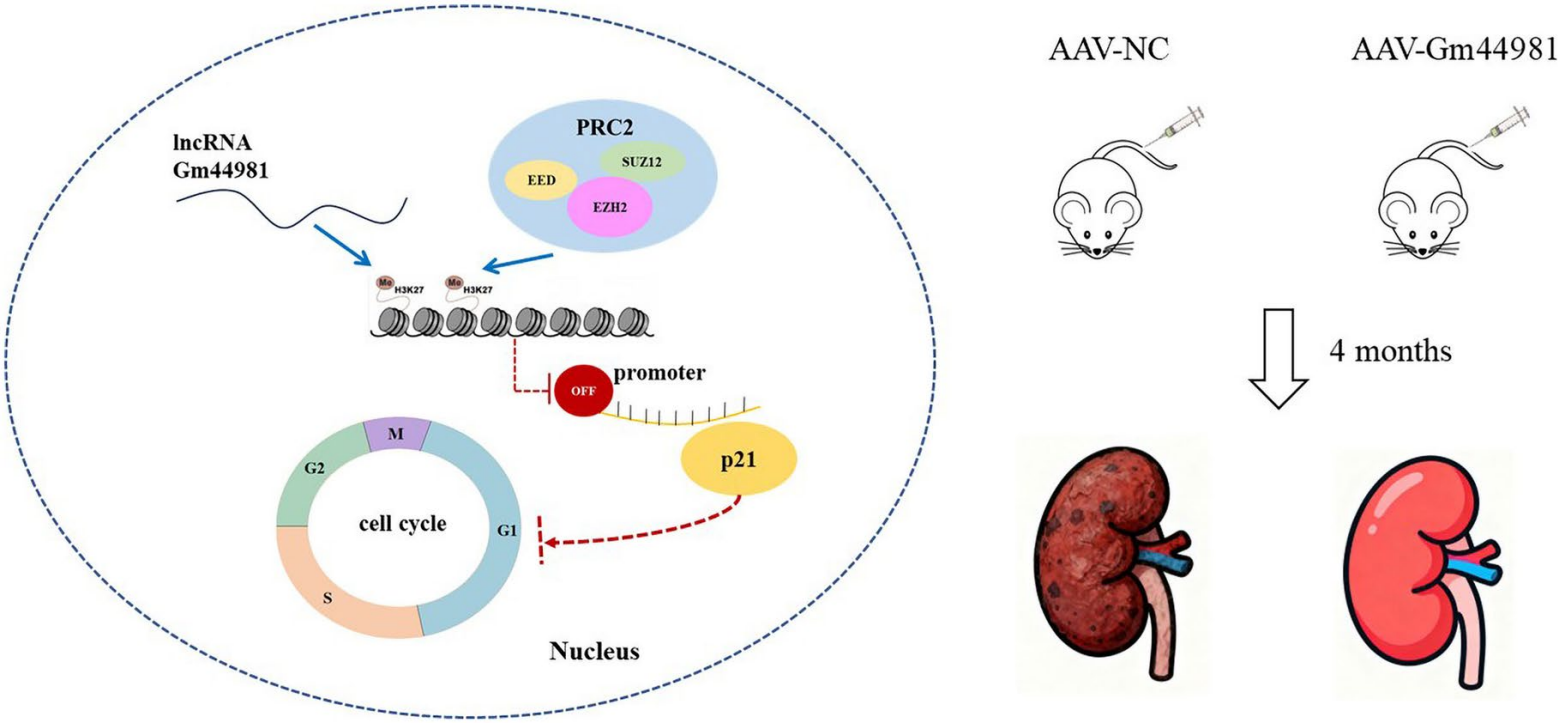
LncRNA Gm44981 modulates EZH2–H3K27me3–p21 axis to suppress mesangial cell senescence and kidney aging.

Together, our study demonstrates that overexpression of *Gm44981* results in increased expression levels of H3K27me3 at the p21 promoter region, thus inhibiting p21 expression, which ultimately inhibits cellular senescence (Figure 6).

## Conclusion

In summary, our findings position *Gm44981* as a key regulator associated with MCs cell cycle progression and inhibition of senescence. This effect correlates with negative regulation of p21 expression and upregulation of EZH2-mediated H3K27me3 methylation, suggesting a potential mechanistic pathway, which could serve as promising targets for kidney aging.

## Supplementary Material

Supplemental Material

Supplemental Material

Supplemental Material

Supplemental Material

## Data Availability

The datasets used and/or analyzed during the current study available from the corresponding author on reasonable request.
