# Supplementary figures and images for: LncRNA Gm44981 modulates EZH2–H3K27me3–p21 axis to suppress mesangial cell senescence and kidney aging
Source: Ren Fail. 2026 Feb 11;48(1):2628471. doi: 10.1080/0886022X.2026.2628471 (PMC12903935; doi:10.1080/0886022X.2026.2628471)

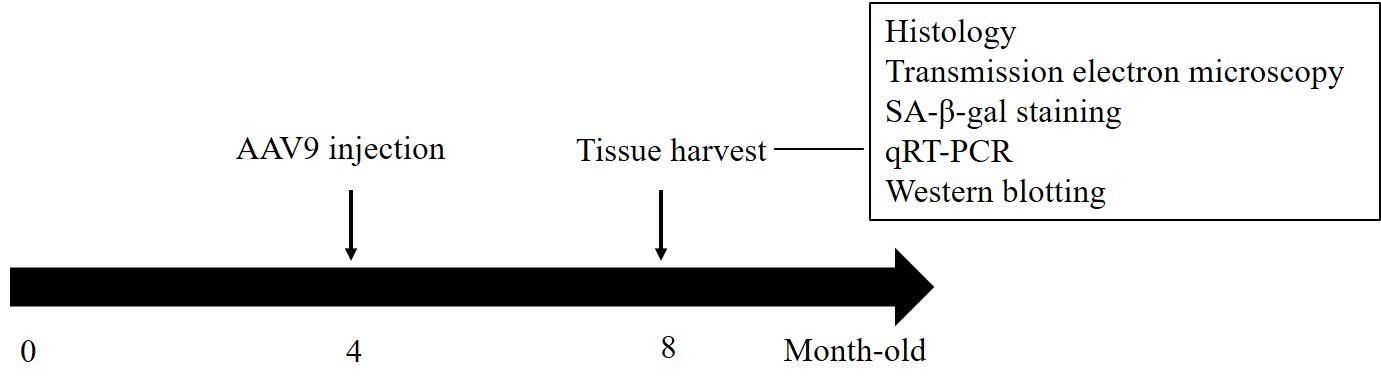

Supplement: Supplemental Material [file IRNF_A_2628471_SM6910.jpg]

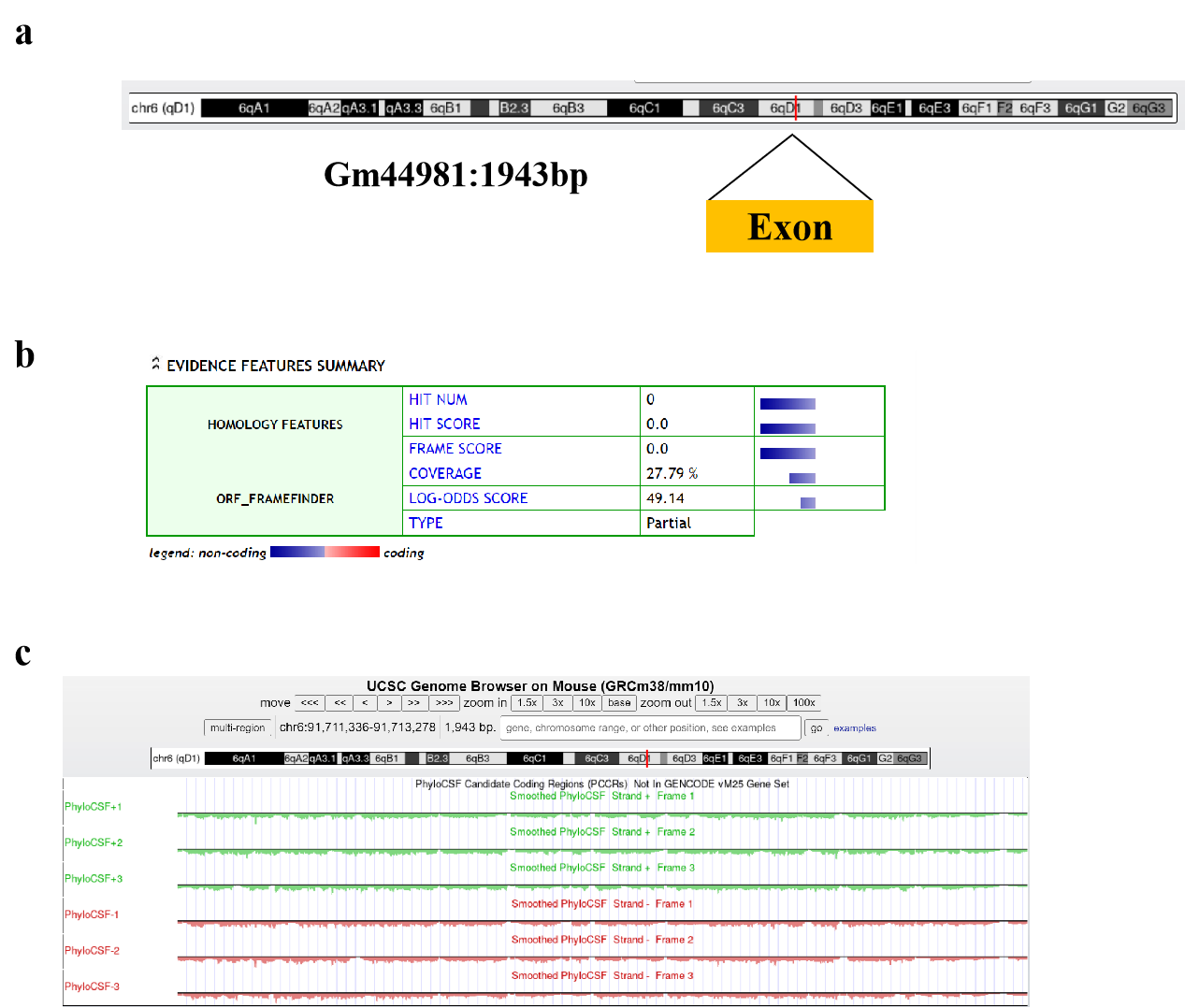

Supplement: Supplemental Material [file IRNF_A_2628471_SM6909.png]

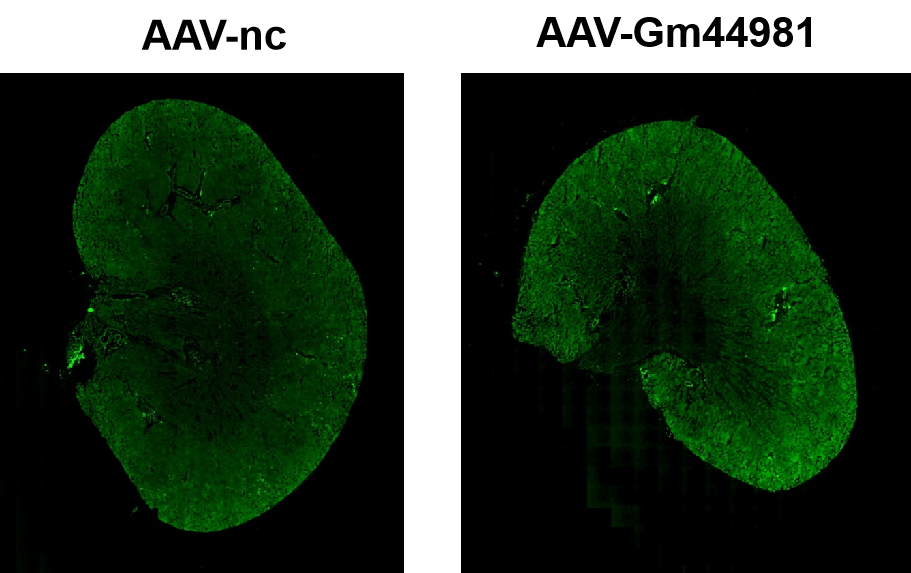

Supplement: Supplemental Material [file IRNF_A_2628471_SM6908.png]

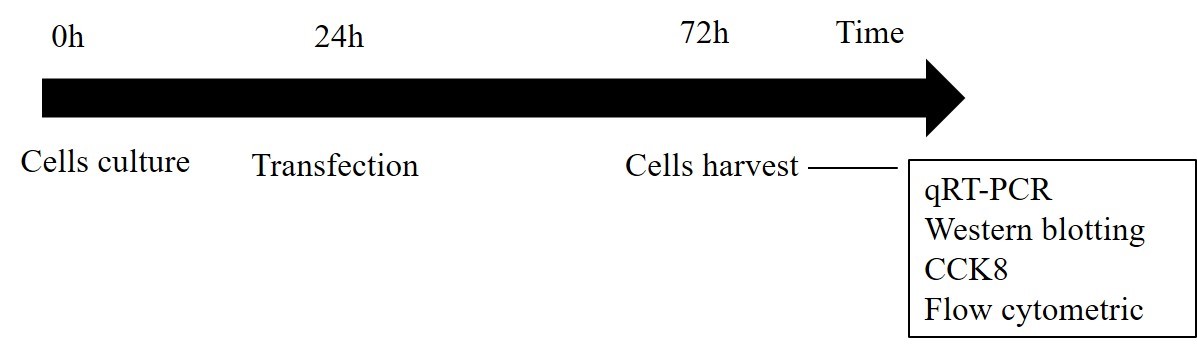

Supplement: Supplemental Material [file IRNF_A_2628471_SM6907.jpg]
